# Core promoter elements are not essential for transcription in mammals

**DOI:** 10.1186/1756-8935-6-S1-P91

**Published:** 2013-03-18

**Authors:** Zohar S Barbash, Jocelvn D Weissman, Jie Mu, Dinah S Singer

**Affiliations:** 1 Experimental Immunology Branch, National Cancer Institute, NIH, Bethesda, MD 20892, USA

## 

Core promoter elements are thought to be the docking site for transcription factors binding, and thereby essential for transcription initiation. The role of core promoter elements has been studied for decades in reporter systems. the central model claim that each cis sequence has an epigenetic role in navigating transcription initiation. We use here the MHC class I gene as model to study transcription in a transgenic mouse.

MHC class I genes are ubiquitously expressed and subject to both tissue-specific and hormonal regulation. The core promoter contains four conventional elements: CCAAT, a TATAA-like element, an Sp1 binding site and a canonical Inr. The in vivo function of these elements was determined by mutating each individually within the context of the native gene.

Surprisingly, none of the elements was essential for transcription since no single mutation eliminated transcription. Indeed, mutation of any one of the elements resulted in increased promoter activity, indicating that these elements function as transcriptional modulators. Further, each of the elements was found to have a distinct function, contributing uniquely to tissue-specificity, hormonal responses or both.

The core promoter elements do not affect start site selection, demonstrating that they do not invoke a cryptic promoter. However, they do modulate relative start site usage. The patterns of chromatin modification reflect the expression status of the different promoters. In tissues where the different promoters constructs support active transcription, histone H3K4 trimethylation is high and H3K9 trimethylation is low. Conversely, H3K9 trimethylation is high and H3K4 trimethylation low across the gene in tissues where the promoter constructs are less active. The wild type promoter is activated by interferon, while the Inr and Sp1 mutants repress transcription in response to interferon treatment.

Finally, the CAAT element was found to have dual function, both as a transcriptional regulator and as a barrier element. The barrier function correlated with the binding of C/EBP and CTCF to the wild type CAAT element and variegated expression across generations when the CAAT element was mutated.

Remarkably, these results demonstrate that none of the elements homologous to canonical core promoter elements are necessary for promoter activity. However, they do contribute to the fine tuning of the tissue specific patterns of expression, extracellular signaling, overall promoter activity and chromatin modifications.

